# *Anagrus dmitrievi* sp. n. (Hymenoptera, Mymaridae), an egg parasitoid of *Zyginidia eremita* (Hemiptera, Cicadellidae), a pest of maize in Xinjiang, China

**DOI:** 10.3897/zookeys.736.20883

**Published:** 2018-02-08

**Authors:** Qin Li, Hongying Hu, Serguei V. Triapitsyn, Long Yi, Jiaxiong Lu

**Affiliations:** 1 College of Life Science and Technology, Xinjiang University, Urumqi, Xinjiang, 830046, China; 2 Entomology Research Museum, Department of Entomology, University of California, Riverside, California, 92521, USA

**Keywords:** China, corn three-spotted leafhopper, egg parasitoid, maize, Mymaridae, taxonomy

## Abstract

A new Palaearctic species of *Anagrus* Haliday (Hymenoptera, Mymaridae), A. (Anagrus) dmitrievi Triapitsyn & Hu, **sp. n.**, is described, diagnosed, and illustrated from Xinjiang Uyghur Autonomous Region of China. It was reared from parasitized eggs of the leafhopper Zyginidia (Zyginidia) eremita Zachvatkin (Hemiptera, Cicadellidae) on leaves of maize. A key to the 21 named species of *Anagrus* known from China is provided. *Zyginidia eremita* is an important economic pest in Xinjiang, occurring in approximately 90% of maize fields. The phenology and life cycle of *Z. eremita* is summarized. Parasitism of its eggs by *A. dmitrievi* in maize fields was 12–75 % (average about 38 %), thus making it a rather effective natural enemy.

## Introduction

The Palaearctic leafhopper *Zyginidia eremita* Zachvatkin (Hemiptera, Cicadellidae, Typhlocybinae), sometimes commonly called the corn three-spotted leafhopper (Yu et al. 1995), is widely distributed in Xinjiang Uyghur Autonomous Region of China. It can cause significant damage to the cultivated crops such as corn, wheat, etc. (Poaceae). The species is also known from Serbia, Ukraine, the European part of Russia, Georgia, Azerbaijan, Uzbekistan, and Kazakhstan (Dmitriev 2017). In Xinjiang, this leafhopper is an important economic pest of maize (Yu et al. 2001; Zhang et al. 2013; Qu et al. 2016). It was previously reported there as *Zygina salina* Mitjaev (Yu et al. 1995; Qu et al. 2000; Yu et al. 2001; Yi et al. 2013; Zhang et al. 2013; Qu et al. 2016), a synonym of *Z. eremita*. Like some other leafhoppers, *Z. eremita* was reported to be able to transmit unidentified plant viruses that cause unnamed diseases which leave spots or scorches on the leaves. It can also cause maize plants to under-produce or even kill them (Yu et al. 2001).

An unidentified species of *Anagrus* Haliday (Hymenoptera, Mymaridae) was collected, with numerous individuals of *Z. eremita*, by sweeping in maize fields in Jimsar and Mulei counties of Xinjiang (Yi et al. 2013). However, even though Yi et al. (2013) reported a positive correlation between this species [as *Anagrus* sp(p). and *A. breviphragma* Soyka] and *Z. eremita* [as *Zygina salina*], a definite host-parasitoid association between them was not established until the fourth author first reared this *Anagrus* sp. (Fig. 4B, C) from parasitized eggs of *Z. eremita* on maize leaves in 2012. Specimens of the parasitoid were then sent to the third author, who determined it as belonging to an undescribed species. Here we describe and illustrate it. A scientific name for this egg parasitoid is needed for forthcoming publications on its biological traits as well as integrated control measures against it, which may include enhancement of natural control. The presented information may also be useful for integrated pest management research in other countries of Eurasia where *Z. eremita* occurs in the fields of economically important poaceous crops.

## Materials and methods

### Taxonomic study on the egg parasitoid

Selected specimens of both sexes of the egg parasitoid were dissected and slide-mounted in Canada balsam, examined under a Zeiss Axioskop 2 plus compound microscope, and photographed using the Auto-Montage system; the photographs were then retouched where necessary using Adobe Photoshop.

Terms used for morphological features are those of Gibson (1997). All measurements were taken from slide-mounted specimens, unless stated otherwise, and are given in micrometers (µm) as length or, for the wings, as length:width. Abbreviations used are:

**F** funicle segment of the female antenna or flagellomere of the male antenna;

**mps** multiporous plate sensillum or sensilla on the antennal flagellar segments (= longitudinal sensillum or sensilla or sensory ridge(s)).

The following collection acronyms are used:

**CNC** Canadian National Collection of Insects, Arachnids and Nematodes, Ottawa, Ontario, Canada;

**ICXU** Insect Collection of College of Life Science and Technology, Xinjiang University, Urumqi (Ürümqi), Xinjiang, China;

**INHS** Illinois Natural History Survey, Champaign, Illinois, USA;

**UCRC** Entomology Research Museum, University of California, Riverside, California, USA.

### Collecting leafhoppers and their egg parasitoids

Leafhoppers had been initially collected by sweeping in the maize fields in Anningqu (Urumqi), Turpan, Ili, Jimsar, Qitai, and Mulei in Xinjiang from April to September each year during 2010–2012; all the specimens were taken to the laboratory for rearing and identification. Several maize fields that had abundant *Z. eremita* populations were then chosen for the further two-year survey, mainly in Anningqu (43.9507°N, 87.4713°E, 582 m), relatively close to Xinjiang University in Urumqi so convenient for collecting both the leafhopper host and its egg parasitoids. Additional rearing of the egg parasitoids and collections of the host leafhoppers were conducted by the fifth author during 2013 corn growing season.

Field samples were taken every 15 days mainly in maize fields and occasionally also in wheat fields. Occurrence, population density, oviposition, and overlaps among the generations of *Z. eremita* were recorded, and damage to the crops by this pest was assessed. From June to September 2012, field samples were collected by sweeping with a net (200 mesh size) every five days; each time we swept for 45 minutes. Adult leafhoppers were collected by aspirator into ventilated containers supplied with maize leaves. An experimental group comprised 12–16 adult leafhoppers of the same morphospecies per container; these were then transported to the laboratory. We also searched in the field for *Z. eremita* eggs imbedded in maize leaves, either by a naked eye or using a portable magnifying glass; once found, the entire maize leaves with the leafhopper eggs were cut and placed into mesh bags.

Both the adult leafhoppers and nymphs were counted for statistical analysis. Maize leaves containing leafhopper eggs were divided into groups, each counted and marked under a microscope, placed in glass Petri dishes (95 mm diameter) or in glass vials (200 mm length, 40 mm diameter). The containers were kept at a suitable humidity by putting a cotton ball dipped in with pure water, and sealed by gauze in order to provide air. Then the vials were placed in an incubator with constant temperatures of either 26 °C or 30 °C and relative humidity of 35 %.

Parasitoids emerging from the parasitized leafhopper eggs were preserved in 75 % ethanol and kept in a refrigerator at +4°C until used for identification. Voucher specimens from this study (both of leafhoppers and their egg parasitoids) are deposited mainly in ICXU, while some of them were also deposited in UCRC.

## Taxonomy

### Anagrus (Anagrus) dmitrievi

Taxon classificationAnimaliaHymenopteraMymaridae

Triapitsyn & Hu sp. n.

http://zoobank.org/038E4562-4E16-4352-BD62-33AD95846C2A

**Figure 1.**
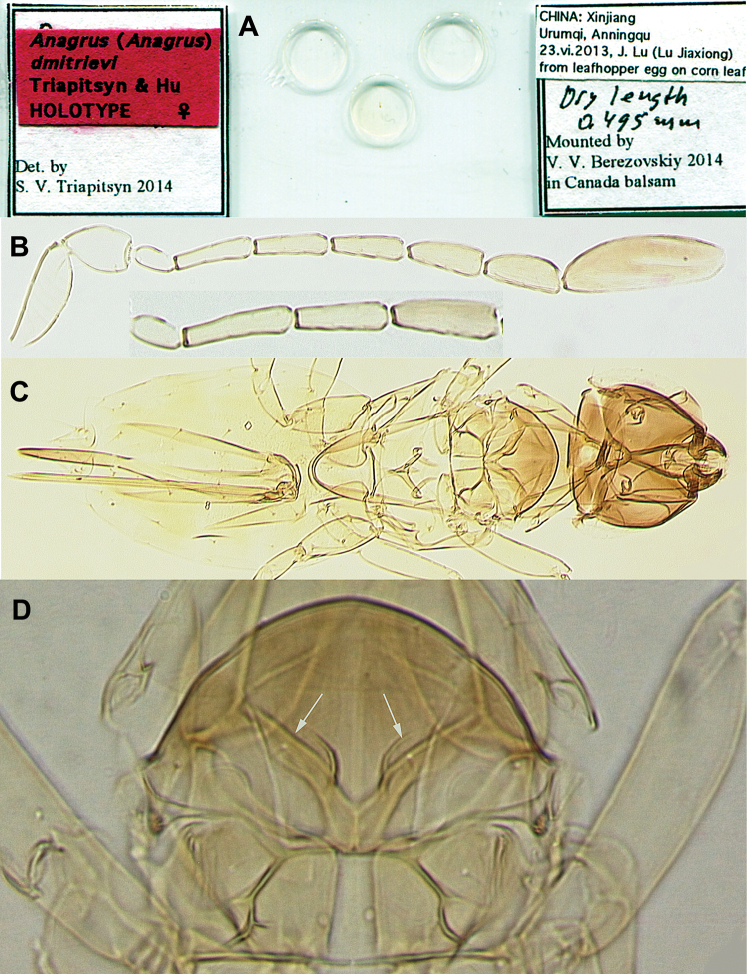
*Anagrus dmitrievi* (female, holotype): **A** slide **B** antennae (complete antenna without mps on F4; F4 of the incomplete antenna with 1 mps) **C** body **D** mesoscutum (arrows pointing to adnotaular setae on its midlobe).

**Figure 2.**
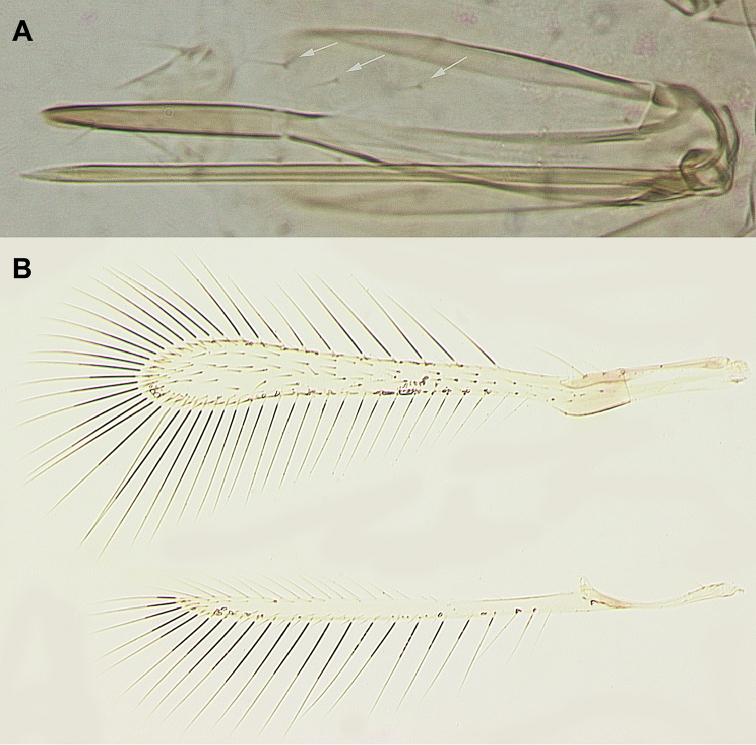
*Anagrus dmitrievi* (female, holotype): **A** ovipositor (arrows pointing to distal setae on one of its external plates, or second valvifers) **B** fore and hind wings.

**Figure 3.**
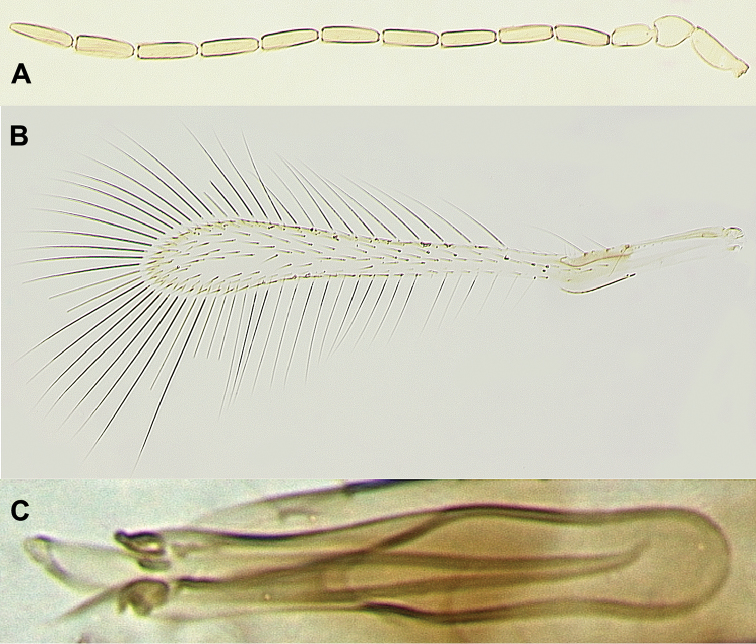
*Anagrus dmitrievi* (male, paratypes): **A** antenna **B** fore wing **C** genitalia.

**Figure 4.**
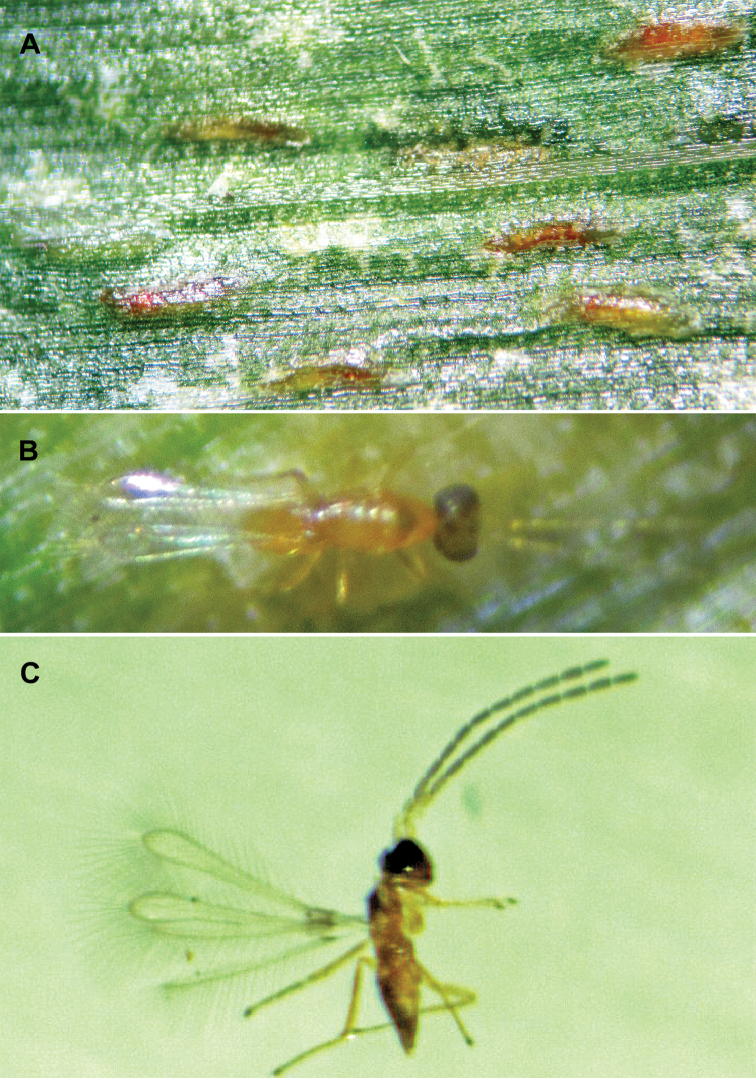
**A** parasitized eggs of *Zyginidia eremita* by *Anagrus dmitrievi* in a maize leaf **B** an adult female of *A. dmitrievi* right after emergence **C** an adult male of *A. dmitrievi*.

Figs 1 , 2 , 3 , 4B, C

Anagrus sp(p).: Yi et al. 2013: 331 (correlation with the host leafhopper in Xinjiang).
Anagrus breviphragma Soyka: Yi et al. 2013: 332 (misidentification).

#### Type material.

Holotype female on slide (Fig. 1A), deposited in ICXU, labeled: 1. “CHINA: Xinjiang, Urumqi, Anningqu, 23.vi.2013, J. Lu (Lu Jiaxiong), from leafhopper egg on corn leaf”; 2. “Dry length 0.495 mm”; 3. “Mounted by V. V. Berezovskiy 2014 in Canada balsam”; 4. [red] “Anagrus (Anagrus) dmitrievi Triapitsyn & Hu HOLOTYPE ♀”; 5. “Det. by S. V. Triapitsyn 2014”. Paratypes: 2 females and 2 males on points [UCRC], and 11 females and 8 males on slides [CNC (1 and 1), ICXU (6 and 4), UCRC (4 and 3), respectively], same data as the holotype (43.9507°N, 87.4713°E, 586 m) except for different body length measurements.

#### Non-type material examined.

Numerous specimens of both sexes in ethanol, deposited in ICXU, reared by L. Yi (Yi Long) from eggs of *Z. eremita* during 2012 (Yi et al. 2013).

#### Diagnosis.

*Anagrus dmitrievi*, sp. n. is characterized by the following unique combination of features: antenna (Fig. 1B) with F1 slightly more than half of pedicel length, F2 longest of funicular segments, F3–F6 subequal in length and slightly shorter than F2, F4 usually without mps but sometimes with one mps (occasionally only on one antenna), F5 with one or two mps, F6 with two mps, and clava with five mps; midlobe of mesoscutum with a pair of adnotaular setae (Fig. 1D); fore wing (Fig. 2B) 9.0–9.1 × as long as wide and its disc with several rows of setae leaving no distinct bare areas in its broadest part; ovipositor exserted beyond apex of gaster by 0.06–0.15 × total own length and 2.1–2.4 × length of protibia, each second valvifer with three distal setae (Fig. 2A).

#### Description.

Female (holotype and paratypes). Body length of dry-mounted, critical point-dried paratypes 400–627 µm (495 µm of the holotype; measurements taken prior to slide-mounting). Head light brown to brown except eyes and ocelli reddish; body mostly yellowish or light brown except anterior half or so of mesoscutum brown, frenum of scutellum white, and propodeum lemon yellow; scape, pedicel and F1 yellow, rest of flagellum brown; legs yellowish, wings hyaline. Antenna (Fig. 1B) with scape 3.5–3.6 × as long as wide, with cross-ridges, 1.7–1.8 × length of pedicel; F1 cylindrical, slightly more than half of pedicel length; F2 longest of funicular segments; F3–F6 subequal in length and slightly shorter than F2; mps on F4 (usually none but sometimes one, occasionally only on one antenna as in the holotype, Fig. 1B); F5 (one or two), and F6 (two); clava with five mps, 3.0–3.3 × as long as wide, about as long as combined length of two preceding segments. Mesosoma shorter than metasoma (Fig. 1C). Midlobe of mesoscutum with a pair of adnotaular setae (Fig. 1D). Fore wing (Fig. 2B) 9.0–9.1 × as long as wide, longest marginal seta 2.8–3.0 × maximum wing width; distal macrochaeta 2.0–2.7 × length of proximal macrochaeta; disc with several rows of setae (two such rows just beyond apex of venation, the row of setae along posterior margin originating behind apex of venation), leaving no distinct bare areas in its broadest part. Hind wing (Fig. 2B) 24–27 × as long as wide, longest marginal seta 6.0–6.5 × maximum wing width; disc mostly bare except for admarginal rows of setae. Ovipositor anteriorly not extending to mesophragma in slide-mounted specimens and posteriorly exserted beyond apex of gaster by 0.06–0.15 × total ovipositor length. Second valvifers (= external plates of ovipositor of authors) (Chiappini 1989; Chiappini et al. 1996; etc.) each with three distal setae (Fig. 2A). Ovipositor 2.1–2.4 × length of protibia (2.3 × in the holotype).

Measurements (µm) of the holotype. Body 627; head 123; mesosoma 209; gaster 307; ovipositor 277. Antenna: scape 73; pedicel 42; F1 24; F2 49; F3 45; F4 45 (48 of the other on which an mps is present); F5 45; F6 48; clava 103. Fore wing 547:61; longest marginal seta 172. Hind wing 517:21; longest marginal seta 136.

Male (paratypes). Body length of the dry-mounted, critical point-dried paratypes (including prior to slide-mounting) 462–594 µm. Similar to female except for the normal sexually dimorphic features such as antenna (Fig. 3A) and genitalia (Fig. 3C), and the following. Body somewhat darker than in female, particularly gaster light brown to brown; fore wing (Fig. 3B) 7.3–8.2 × as long as wide.

#### Remarks.

The new species belongs to the *incarnatus* species group of the nominate subgenus of *Anagrus*, as defined by Chiappini et al. (1996), in whose key it runs to *A. flaveolus* Waterhouse (see below). In Chiappini and Lin (1998), *A. dmitrievi* keys in the same couplet together with *A. striatus* Chiappini & Lin, a completely different species whose female has one mps on F3 and a bare area on the broadest part of the fore wing disc. In Triapitsyn and Berezovskiy (2004), *A. dmitrievi* keys to *A. nigriceps* (Smits van Burgst) in which an mps is present on F3 of the female antenna (Chiappini et al. 1996) whereas it is always absent on that segment in *A. dmitrievi*.

When, as usually, F4 of the female antenna of *A. dmitrievi* lacks an mps, it is most similar to the Nearctic species *A. sophiae* S. Trjapitzin, which, however, has a relatively much longer ovipositor (2.7–3.8 × length of protibia) (Trjapitzin and Strong 1995). When F4 of the female antenna bears an mps (at least on one antenna), it is most similar to the New World species *A. flaveolus*, from which it differs in F5 being about as long as F4 and F6 and also in having the row of setae along the posterior margin and originating behind the apex of venation of the fore wing (whereas in *A. flaveolus*, F5 is always shorter than F4 and F6, even when an mps is present, and the row of setae along the posterior margin of the fore wing does not extend to apex of venation). Those females of *A. dmitrievi* that bear an mps on F4 on both antennae are also somewhat similar to females of the Palaearctic species *A. brocheri* Schulz, whose general body color is much darker (dark brown).

In the world key to the species of *Anagrus* by Triapitsyn (2015), females of *A. dmitrievi* key either to *A. sophiae* (when an mps is present on F4) or *A. flaveolus* (when an mps is absent on F4).

The updated key to the Chinese species of *Anagrus* (below) was modified from Triapitsyn (2015). The previous key (Chiappini & Lin 1998) is missing the five species, besides *A. dmitrievi*, described or recorded since then (A. (Anagrus) fragranticus Triapitsyn, A. (Anagrus) incarnatus Haliday [as A. (Anagrus) breviphragma Soyka], A. (Anagrus) kvas Triapitsyn & Berezovskiy, A. (Anagrus) nigriceps (Smits van Burgst), and A. (Anagrus) turpanicus Triapitsyn & Hu) (Triapitsyn 2003, 2015; Triapitsyn and Berezovskiy 2004; Hu and Triapitsyn 2016). Thus, 21 named species of *Anagrus* are now known from China.

#### Etymology.

The species is named after Dmitry A. Dmitriev (INHS) who kindly identified the leafhopper host of this parasitoid.

#### Host.

The parasitoids of the type series were definitely reared from eggs of *Zyginidia eremita* because we also reared this leafhopper from its unparasitized eggs during the same collecting event; these leafhoppers were later compared with the positively identified specimens.

#### Biology and parasitism.

Leafhopper’s eggs parasitized by *A. dmitrievi* turn dark reddish (Fig. 4A). The optimal constant incubation temperature under laboratory conditions for immature *A. dmitrievi* was 25 °C, at which about 38 % of the eggs hatched. Estimated field parasitism rate of *Z. eremita* eggs by *A. dmitrievi* in Xinjiang was 12–75 % (average about 38 %), thus making it a rather effective natural enemy.

##### Key to females of the Chinese species of *Anagrus*

**Table:** 

1	Ocelli on a stemmaticum	**2**
–	Ocelli not on a stemmaticum (subgenus A. (Anagrella) Bakkendorf)	**4**
2	Mesoscutum without notauli	***Anagrus dalhousieanus* Mani & Saraswat**
–	Mesoscutum with notauli	**3**
3	Frenum of scutellum with triangular paramedial plates widely separated from each other; metafemur short, less than 2 × trochanter length, trochantellus incision almost half way between coxa-trochanter and femur-tibia articulations (subgenus A. (Paranagrus) Perkins)	**7**
–	Frenum of scutellum with triangular paramedial plates very close to each other; metafemur long, more than 2 × trochanter length, trochantellus incision about one third way between coxa-trochanter and femur-tibia articulations (subgenus A. (*Anagrus* Haliday) [*sensu sricto*])	**8**
4	F2 approximately 1.5 × F1 length	**Anagrus (Anagrella) brevis Chiappini & Lin**
–	F2 at least 2.0 × F1 length	**5**
5	Fore wing disc without setae	**Anagrus (Anagrella) albiclava Chiappini & Lin**
–	Fore wing disc with setae	**6**
6	F5 without mps; F3, F4 and F5 together at most as long as clava	**Anagrus (Anagrella) hirashimai Sahad**
–	F5 with 1 mps; F3, F4 and F5 together longer than clava	**Anagrus (Anagrella) semiglabrus Chiappini & Lin**
7	Ovipositor projecting beyond apex of gaster by approx. 1/3 of its total length; ovipositor: protibia ratio at least 3.5	**Anagrus (Paranagrus) perforator (Perkins)**
–	Ovipositor not projecting or at most slightly projecting beyond apex of gaster; ovipositor: protibia ratio at most 2.5	**Anagrus (Paranagrus) optabilis (Perkins)**
8	Clava with 3 mps (*atomus* species group)	**9**
–	Clava with 5 mps (*incarnatus* species group)	**14**
9	F3 with 1 mps	**10**
–	F3 without mps	**12**
10	Fore wing disc without a hairless area in the broadest part	**Anagrus (Anagrus) setosus Chiappini & Lin**
–	Fore wing disc with a distinct hairless area in the broadest part	**11**
11	F4 with 2 mps	**Anagrus (Anagrus) flaviapex Chiappini & Lin**
–	F4 with 1 mps	**Anagrus (Anagrus) frequens Perkins** (part)
12	Fore wing disc without a hairless area in the broadest part	**Anagrus (Anagrus) kvas Triapitsyn & Berezovskiy**
–	Fore wing disc with a distinct hairless area in the broadest part	**13**
13	Fore wing disc with hairless area occupying its whole posterior half; fore wing length: width ratio more than 10.5	**Anagrus (Anagrus) frequens Perkins** (part)
–	Fore wing disc with hairless area only in the broadest part; fore wing length: width ratio at most 10.0	**Anagrus (Anagrus) atomus (Linnaeus)**
14	F1 about as long as pedicel or at most slightly shorter (by less than 0.2 × pedicel length); ovipositor exserted beyond apex of gaster by about 1/3 of its total length	**Anagrus (Anagrus) paranagrosimilis Chiappini & Lin**
–	F1 shorter than pedicel by more than 0.25 × pedicel length; ovipositor exserted beyond apex of gaster by less than 1/3 of its total length or not exserted	**15**
15	Clava about as long as combined length of three preceding funicular segments	**Anagrus (Anagrus) minutus Chiappini & Lin**
–	Clava notably shorter than combined length of three preceding funicular segments	**16**
16	Fore wing at most 6.0 × as long as wide	**Anagrus (Anagrus) fragranticus Triapitsyn**
–	Fore wing at least 7.0 × as long as wide	**17**
17	Fore wing disc with a more or less distinct hairless area in the broadest part	**18**
–	Fore wing disc without a hairless area in the broadest part	**20**
18	Mesoscutum with adnotaular setae	**Anagrus (Anagrus) striatus Chiappini & Lin**
–	Mesoscutum without adnotaular setae	**19**
19	F2 the longest funicular segment; ovipositor at least 2.7 × length of protibia	**Anagrus (Anagrus) incarnatus Haliday**
–	F2 shorter than F4, F5, or F6; ovipositor at most 2.4 × length of protibia	**Anagrus (Anagrus) turpanicus Triapitsyn & Hu**
20	Mesoscutum without adnotaular setae	**Anagrus (Anagrus) nilaparvatae Pang & Wang**
–	Mesoscutum with adnotaular setae	**21**
21	F3 without mps	**Anagrus (Anagrus) dmitrievi Triapitsyn & Hu, sp. n.**
–	F3 with at least 1 mps	**Anagrus (Anagrus) nigriceps (Smits van Burgst)**

##### Notes on the leafhopper host of *Anagrus dmitrievi*

**Identification of the host leafhopper**

The corn three-spotted leafhopper, collected on 7.viii.2012 by J. Lu (Lu Jiaxiong) and L. Yi (Yi Long) on maize plants at the same locality as the type series of *A. dmitrievi*, were identified by Dmitry A. Dmitriev as Zyginidia (Zyginidia) eremita (voucher specimens in INHS and additional 2 females in UCRC). This was the most abundant leafhopper species feeding on maize plants in Anningqu. Another rather common leafhopper on maize in Xinjiang was *Cicadella viridis* (Linnaeus) (Yi et al. 2013).

**Figure 5.**
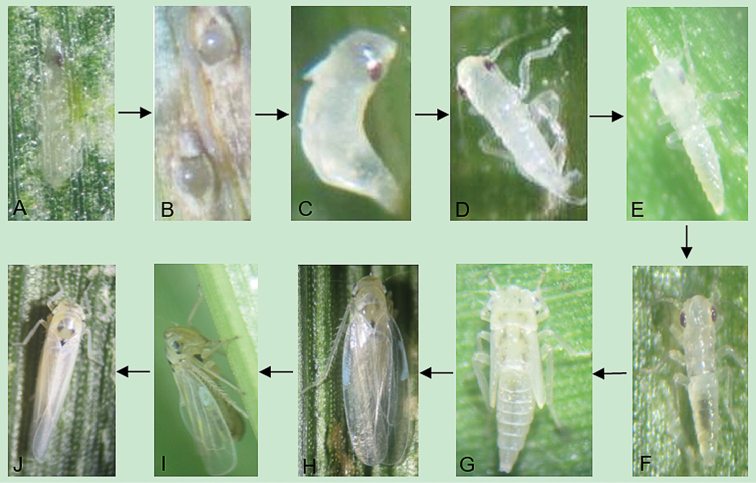
Life cycle of *Zyginidia eremita*: **A, B**, egg **C–G** nymphs **H–J** adults.

Adult *Z. eremita* (Fig. 5H–J) have a characteristic grayish white color; light brown markings decorate the costal margin of vertex. The pronotum is transparent, and three equal-sized oval black spots are present on the adults’ mesoscutum. The fore and hind wings are crystal white, and the abdomen has black, transverse, dorsal bands. The average length of adult *Z. eremita* is 2.6–2.8 mm.

**Records, phenology, and life cycle of *Z. eremita***

**Figure 6.**
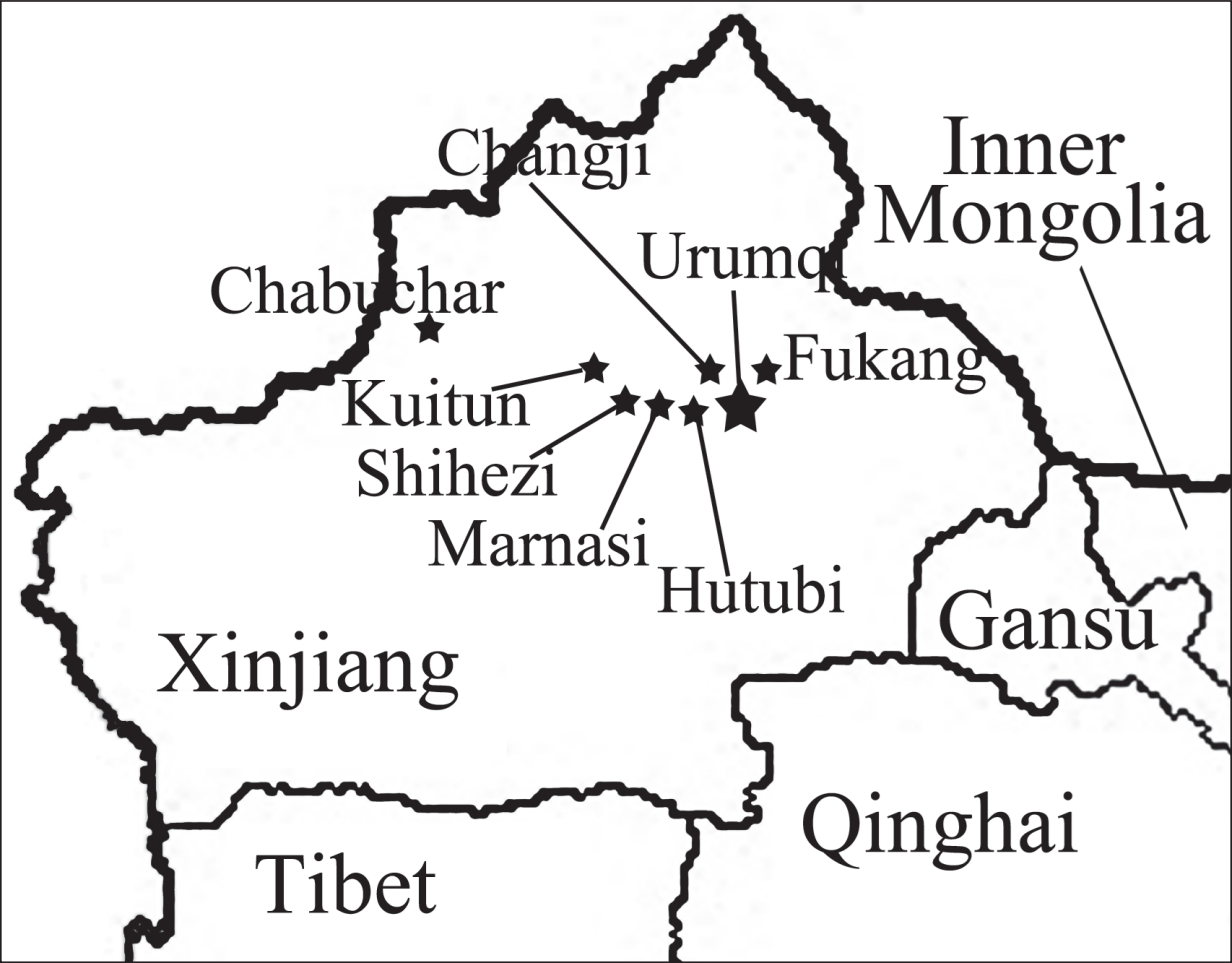
Records of *Zyginidia eremita* in Xinjiang. The large star denotes the type locality of *Anagrus dmitrievi*.

*Zyginidia eremita* has quite frequent records in Xinjiang (Fig. 6). It occurred in almost every maize field sampled. Only 5–10 % of the maize fields had a relatively sparse population of *Z. eremita*. The highest population densities observed were in Jimsar, Qitai, Mulei, Changji, and Urumqi.

In Xinjiang (eastern Xinjiang: Jimsar, Qitai, Mulei, Balikun, and Hami; north-central Xinjiang: Ili, Turpan, Korla, and Urumqi (Anningqu)), *Z. eremita* has three generations per year and these overlap during the warm months. According to Yu et al. (1995), adult leafhoppers of this species overwinter in winter wheat or in poaceous weeds. But according to Yu et al. (2001), adults also overwinter in surrounding trees, although no details exactly where they hide were provided by these authors.

Adult leafhoppers start to emerge from their overwintering shelters in late April; in about mid-May, at least some of them apparently move to winter and spring wheat (Yu et al. 1995). The first generation of nymphs appears on wheat plants in late May. In early June, the first generation of adult leafhoppers migrates to maize fields and feeds on the seedlings. Their peak oviposition period occurs in late June.

The leafhopper species collected by J. Lu on 13.vi.2014 in the wheat field in Anningqu was, however, not *Z. eremita* but Macrosteles (Macrosteles) alpinus (Zetterstedt) [2 females and 2 males in UCRC, determined by D. A. Dmitriev].

Females of *Z. eremita* prefer relatively mature leaves of maize plants for oviposition. Most eggs are laid in the leaves near the ground, very rarely in the upper leaves on a plant. Transparent oval eggs of the corn three-spotted leafhopper are usually laid into the leaf tissue near the bottom of a leaf close to the middle vein.

In early July, the second generation of nymphs hatches, and within about ten days their population density becomes very high. The second generation of adults oviposits in maize leaves in late July and early August. Peak abundance of the third generation nymphs is in mid to late August. Adult leafhoppers of the third generation gradually move in the fall to winter wheat (Yu et al. 1995) or grass (Yu et al. 2001).

Life history and phenology of *Z. eremita* are summarized in Table 1, and its life cycle is shown in Fig. 5.

**Table 1.** Life history of *Zyginidia eremita* in Anningqu, Urumqi, Xinjiang. ●Egg ▲ Nymph ★ Adult.

Month	April	May	June	July	August	September	October	November to March
Period	Early Mid Late	Early Mid Late	Early Mid Late	Early Mid Late	Early Mid Late	Early Mid Late	Early Mid Late	Early Mid Late
1^st^ genera-tion	★ ● ●	● ● ●						
		▲ ▲	▲ ▲					
		★	★ ★ ★	★				
2^nd^ genera-tion			● ●	● ● ●				
				▲ ▲ ▲	▲ ▲			
				★	★ ★ ★	★		
3^rd^ genera-tion					● ● ●	● ●		
					▲ ▲	▲ ▲ ▲		
					★	★ ★ ★	★ ★ ★	★ ★ ★

## Discussion

The only other available records of egg parasitoids of *Zyginidia* spp. are those of *Anagrus atomus* (Linnaeus) from Z. (Zyginidia) pullula (Boheman) on maize in Italy (Vidano and Arzone 1988), from Z. (Zyginidia) scutellaris (Herrich-Schäffer) on maize in France (della Giustina and Caruhel 1989), and from *Z. sohrab* Zachvatkin on maize in Turkey (Mutlu and Sertkaya 2015), as well as of *Lymaenon litoralis* (Haliday) (Mymaridae) from *Z. sohrab* on wheat in Iran (Fallahzadeh and Huber 2011). Baquero and Jordana (1999) reported that large numbers of *A. atomus*, an egg parasitoid of *Z. scutellaris*, were captured in Navarre, Spain, where it is the most abundant leafhopper in maize fields.

The following specimens of *Anagrus atomus* were identified by the third author: Turkey, Diyarbakir Province, Bismil-Diyarbakir, 10.viii.2009, Ç. Mutlu, from eggs of *Zyginidia* sp. on maize, *Zea mays* [4 females and 5 males, UCRC].

## Supplementary Material

XML Treatment for Anagrus (Anagrus) dmitrievi
